# Harvesting the low-hanging fruit? Comparative assessment of intravenous to oral route antimicrobial conversion policy implementation

**DOI:** 10.1017/ice.2022.158

**Published:** 2023-06

**Authors:** Rebekah W. Moehring, Angelina Davis, Elizabeth Dodds Ashley, April P. Dyer, Richard H. Drew, Yuliya Loknyghina, Melissa D. Johnson, Travis M. Jones, S. Shaefer Spires, Daniel J. Sexton, Deverick J. Anderson

**Affiliations:** 1 Duke Center for Antimicrobial Stewardship and Infection Prevention, Duke University Medical Center, Durham, North Carolina; 2 Duke Antimicrobial Stewardship Outreach Network, Duke University Medical Center, Durham, North Carolina; 3 Duke Department of Biostatistics, Duke University Medical Center, Durham, North Carolina

## Abstract

Policies that promote conversion of antibiotics from intravenous to oral route administration are considered “low hanging fruit” for hospital antimicrobial stewardship programs. We developed a simple metric based on digestive days of therapy divided by total days of therapy for targeted agents and a method for hospital comparisons. External comparisons may help identify opportunities for improving prospective implementation.

Policies that promote conversion of antibiotics from intravenous to oral route administration are considered (ie, intravenous [IV] to oral [PO] conversion protocols) are considered a simple, straightforward policy-level intervention for hospital antimicrobial stewardship programs (ASPs), sometimes referred to as “low-hanging fruit.”^1–3^ Such policies target patients receiving agents with high oral bioavailability. These protocols typically require pharmacists to use simple eligibility criteria to contact prescribing clinicians to recommend oral conversion or may allow automatic therapeutic interchange. Potential benefits of IV-to-PO conversion protocols include reductions in pharmacy and hospitalization costs, reduced use of intravenous lines and lowered risk of line-related complications, improved patient comfort, and reduced effort from nurses. These benefits accrued while maintaining treatment efficacy.^4–6^

Although many hospitals have IV-to-PO protocols in place, few studies have assessed whether their institution is capitalizing on opportunities for IV-to-PO conversions.^7^ Additionally, some hospitals struggle to consistently perform IV-to-PO conversions due to competing priorities. We developed a simple calculation of digestive days of therapy (dDOT) divided by total days of therapy (tDOT) to assess how implementation of IV-to-PO conversion policies varied across hospitals, units, and targeted agents. We also developed reports with comparisons to other network hospitals to help assess and refine protocol implementation.

## Methods

We performed a retrospective analysis of existing antimicrobial use data. Electronic medication administration record (eMAR) data from adult and pediatric admissions were extracted from the Duke Antimicrobial Stewardship Outreach Network (DASON) central database from July 2018 through June 2019 for 16 community hospitals.^8^ Analyses included inpatient units but excluded outpatient areas, emergency departments, and procedural units as well as inhaled or topical routes. We defined targeted agents as highly bioavailable antimicrobials typically included in IV-to-PO conversion policies (Table 1). We calculated dDOT as the number of DOT administered via an oral, tube, or per rectum route consistent with National Healthcare Safety Network (NHSN) methods.^9^ We defined tDOT as the number of DOT administered via an intravenous or a digestive route. The dDOT/tDOT process metric was the dDOT divided by the tDOT, calculated for each encounter that used a targeted agent. Mean dDOT/tDOT for all targeted agents together and each agent separately were graphed to show the distribution among hospitals and rank, with highest dDOT/tDOT considered rank first. To demonstrate the value of network comparisons, we have provided an example hospital-specific report (Supplementary Material online).

**Table 1.** Observed Digestive Days of Therapy Divided by Total Days of Therapy (dDOT/tDOT) Estimates and Characteristics of Encounters with Targeted Agents among 16 Hospitals

Variable	No. (%) of Courses	dDOT, No.	tDOT, No.	dDOT/tDOT Per Encounter, Mean (SD)
All agents	50,344 (100)	55,868	156,233	0.36 (0.45)
Azithromycin	12,599 (25)	13,748	35,250	0.38 (0.45)
Ciprofloxacin	6,323 (13)	6,749	18,800	0.38 (0.46)
Doxycycline	6,266 (13)	14,065	21,704	0.66 (0.45)
Linezolid	1,632 (3)	2,537	6,921	0.40 (0.47)
Metronidazole	9,234 (18)	6,840	33,970	0.20 (0.36)
Respiratory fluoroquinolone^a^	14,180 (28)	11,415	38,860	0.29 (0.42)
Voriconazole^b^	110 (<1)	514	728	0.66 (0.43)
**Age**				
<18 y	253 (<1)	275	659	0.40 (0.47)
18–39 y	4,468 (9)	3,836	12,485	0.32 (0.44)
40–65 y	18,359 (37)	19,937	57,519	0.35 (0.44)
>65 y	26,192 (53)	30,636	82,248	0.37 (0.45)
**Sex**				
Female	28,611 (57)	31,044	87,896	0.35 (0.45)
Male	21,733 (43)	24,824	68,337	0.36 (0.45)
**Race/Ethnicity**				
**White**	32,149 (66)	36,395	99,832	0.36 (0.45)
Black	13,722 (28)	15,189	43,129	0.36 (0.45)
Hispanic	217 (<1)	278	570	0.51 (0.47)
Asian	287 (<1)	266	880	0.32 (0.43)
Native American	1,312 (3)	1,267	3,745	0.33 (0.45)
Other	677 (1)	628	2,091	0.28 (0.42)
Unknown	1,980			
**Elixhauser comorbidity score**				
0	5,744 (12)	4,462	12,192	0.38 (0.47)
1–2	11,310 (23)	11,975	32,415	0.37 (0.46)
3–4	15,926 (32)	18,464	49,175	0.37 (0.45)
5–6	11,125 (22)	13,589	38,252	0.35 (0.45)
>7	5,966 (12)	7,104	23,314	0.29 (0.42)
**Length of stay**				
≤2 d	3,647 (7)	1987	5092	0.38 (0.48)
3–7 d	32,098 (64)	30,614	85,165	0.36 (0.45)
>7 d	14,536 (29)	23,187	65,407	0.35 (0.44)
**Unit type on day 1 of therapy**				
Orthopedic ward	123 (<1)	267	316	0.83 (0.35)
Telemetry ward	5,023 (10)	7,465	15,462	0.48 (0.48)
Hematology/Oncology ward	1,213 (2)	1,863	3,979	0.46 (0.47)
Surgical cardiothoracic critical care	40 (<1)	50	98	0.42 (0.48)
Gynecology ward	33 (<1)	29	79	0.40 (0.47)
Medical/Surgical ward	12,730 (26)	14,469	37,590	0.38 (0.46)
Pediatric medical/Surgical ward	300 (<1)	336	839	0.39 (0.46)
Surgical ward	6,961 (14)	7,282	20,817	0.36 (0.46)
Medical ward	14,018 (28)	14,768	41,586	0.36 (0.45)
Adult step-down unit	2,975 (6)	3,405	9,685	0.35 (0.44)
Mixed acuity	813 (2)	779	2,511	0.31 (0.43)
Medical/Surgical critical care	6,115 (12)	5,155	23,271	0.19 (0.36)
**Hospital size in thousand annual patient days**				
<30 d	7,664 (15)	8,698	23,514	0.37 (0.45)
30–70 d	28,099 (56)	32,939	87,906	0.37 (0.46)
>70 d	14,581 (29)	14231	44,813	0.32 (0.44)

a Respiratory fluoroquinolone includes both levofloxacin and moxifloxacin.

b Voriconazole used at 14 of 16 hospitals.

We used negative-binomial regression models to produce an estimated dDOT/tDOT for each hospital and antimicrobial agent. Models included the following covariates: hospital size, length of stay, age, unit type, Elixhauser comorbidity score,^10^ and month. The all agents model also included adjustment for agent. Due to our sample size of 16 hospitals, network estimates were subject to outlier effects. We excluded the target hospital’s data when modeling expected dDOT/tDOT for that hospital to address this limitation. For example, in the model designed to evaluate “hospital X,” data from hospital X were excluded from data sets, then model parameters were produced based on other hospitals’ data. Next, covariates from hospital X were used to calculate an estimated dDOT/tDOT outcome from the model. To create a margin of error range for hospital-specific estimates, we calculated the median absolute difference of other hospitals’ observed rates from hospital X’s estimated rate and added and subtracted to create upper and lower bounds. Hospital-specific reports included graphs of dDOT/tDOT by month and tables describing rates among units. Unit was defined on day 1 of the course of a targeted agent. Reports also provided the observed mean among units of the same NHSN type within the network (Supplementary Material online). This quality-improvement activity was reviewed by the Duke University Institutional Review Board and was deemed exempt. Analyses were performed using SAS version 9.4 software (SAS Institute, Cary, NC).

## Results

**Fig. 1.**
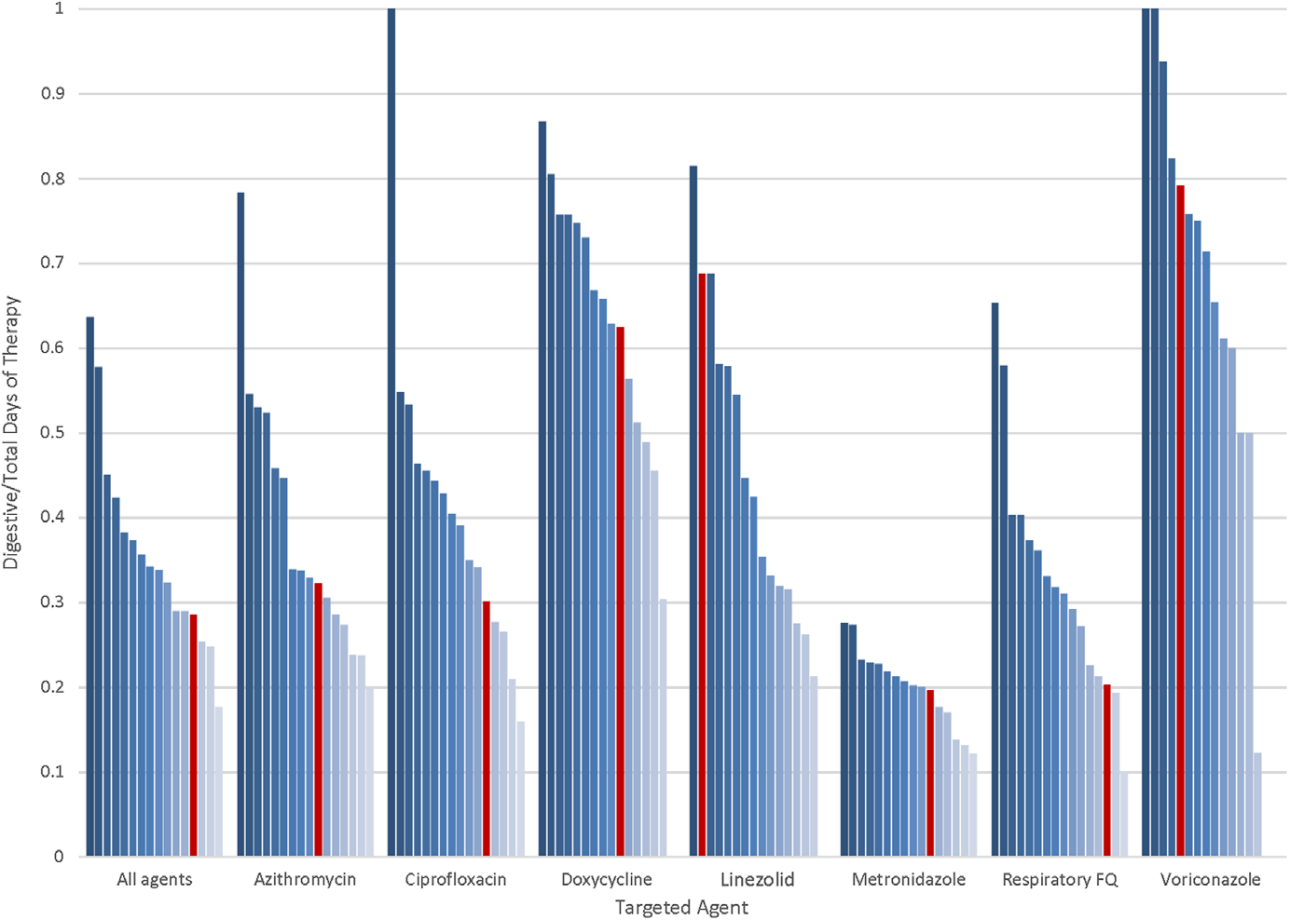
Observed digestive days of therapy divided by total days of therapy (dDOT/tDOT) by targeted agent and hospital comparison. Observed distributions by agent and hospital. Each bar represents an individual hospital. Red bars represent estimates from a specific hospital to aid in comparison to other network hospitals. Note. FQ, fluoroquinolone, which includes levofloxacin and moxifloxacin.

In total, 50,344 courses of a targeted agent were prescribed during 40,682 hospital encounters, totaling 156,233 annual DOT among 16 hospitals. The dDOT totaled 55,868, with mean dDOT/tDOT of 0.36 per course. The DOT were frequently either all digestive (ie, dDOT/tDOT = 1) or fully intravenous (ie, dDOT/tDOT = 0); thus, standard deviations were wide. Patterns of variability emerged when data were aggregated on the hospital and agent level (Fig. 1). The highest dDOT/tDOT values were observed for doxycycline and voriconazole and the lowest for metronidazole. Hospital-level variation was wide for most agents, except metronidazole. Encounter characteristics associated with variation in dDOT/tDOT included length of stay, comorbidity score, and unit type (Table 1). Intensive care units had lowest dDOT/tDOT. Hospital-specific estimates based on negative-binomial modeling and network margins of error, provided a range from which individual hospitals could compare their observed estimates of dDOT/tDOT (Supplementary Material online).

## Discussion

We used a simple, dDOT/tDOT process metric for hospital- and network-comparisons to provide insight on opportunities to promote IV-to-PO conversions. We observed wide variation in dDOT/tDOT among hospitals, at least some of which was due to factors such as targeted agent, type of unit, and comorbidity score. We developed a method to compare observed estimates and adjusted estimates with a margin of error to assist in tracking prospective implementation of IV-to-PO conversion policies.

Hospital IV-to-PO conversion policies have been used for decades as a practical, pharmacy-led intervention to recoup costs and improve care. However, the wide variation detected in this study suggests that implementation of such policies may be inconsistent. Further, IV-to-PO conversion criteria vary among hospitals. Policies with more restrictive criteria provide less opportunities to perform conversions, and comparative data may reveal a need to update hospital policy. Review of local antimicrobial use data can identify opportunities for improvement and help motivate staff to incrementally improve. However, analytic methods to provide such feedback for IV-to-PO conversion policies have not been widely shared, despite suggestions that such processes should be tracked.^3^

Prior investigators have employed process metrics for identifying opportunities for IV-to-PO conversion policies^4,5^ but few have provided hospital-level comparisons.^2^ In practice, IV-to-PO conversions are on a lengthening list of priorities for clinical pharmacists to navigate on a daily basis. When balancing priorities, external comparisons may help bring this need to the attention of pharmacy departments and ASPs. A similar, unit-level metric of dDOT/tDOT could be calculated using aggregate data available through the NHSN antibiotic use option, potentially with external comparisons, for hospitals in the United States to track progress.

This study had several limitations. The study population included 16 hospitals in the southeastern United States that participate in a stewardship network, which affects generalizability.^8^ Furthermore, we used patient-encounter level data to calculate the process metric and evaluate encounter-specific factors for use in modeling adjustments. Some hospitals or systems may only have unit-level DOT estimates readily available. Route, however, is a standardized element in external reporting to the NHSN; unit- or hospital-level estimates could provide useful information even without adjustments for other factors. Our definition for unit may have led to misclassification of digestive DOT to the ICU rather than where the patient subsequently transferred. Thus, interpretation of the ICU-level estimates should be inclusive of practice for both ICUs and transfers. We used adjustment factors readily available in our limited data set; other potential covariates of case mix could improve comparisons. Finally, this was a noninterventional descriptive analysis. Next steps could include assessing whether data feedback encourages local improvement efforts.

In conclusion, dDOT/tDOT is a simple metric that can be used to evaluate implementation of IV-to-PO conversion policies. We observed wide variation by hospital and developed a method by which comparisons could help identify improvement opportunities.

## Supplementary material

For supplementary material accompanying this paper visit https://doi.org/10.1017/ice.2022.158. click here to view supplementary material
