# Supplementary material for: Harvesting the low-hanging fruit? Comparative assessment of intravenous to oral route antimicrobial conversion policy implementation
Source: Infect Control Hosp Epidemiol. 2022 Jul 15;44(6):954–8. doi: 10.1017/ice.2022.158 (PMC10273142; doi:10.1017/ice.2022.158)
Supplement: Supplementary file 1 [file S0899823X22001581sup001.pdf]

Supplement 1. Example Hospital-Specific Report

DUKE ANTIMICROBIAL STEWARDSHIP OUTREACH NETWORK

Hospital Example

Digestive/Total Days of Therapy, June 2018 to July 2019

Summary

A total of 3,725 courses and 12,332 days of antimicrobials typically targeted for intravenous to oral conversions occurred at Hospital Example from June 2018 to July 2019. A total of 3,725 of these antimicrobial days were via digestive administrations. Many of these courses of antimicrobials could represent opportunities to avoid unnecessary intravenous antimicrobials due to their high bioavailability in oral formulation. Early switch to oral formulation could result in cost benefit as well as impact patient outcomes (e.g. line use, length of stay).

Hospital Example demonstrated a mean digestive/total days of targeted antimicrobials of 0.42, and was ranked 4 of 16 DASON hospitals. This rank improved since the last reporting period (mean 0.38, rank 6 of 16.) Time trends continue to show improvement starting in mid-2017 and continuing through 2019. One month the estimate was close to 0.50. Hospital Example was near to the predicted mean based on other hospitals' data and adjustment factors. Areas to focus future investigation and efforts to improve intravenous to oral switches include the following:

- Agents: ciprofloxacin, metronidazole
- Units: 5N, 4N

Figure 1. Mean Digestive/Total by Agent, All Hospitals

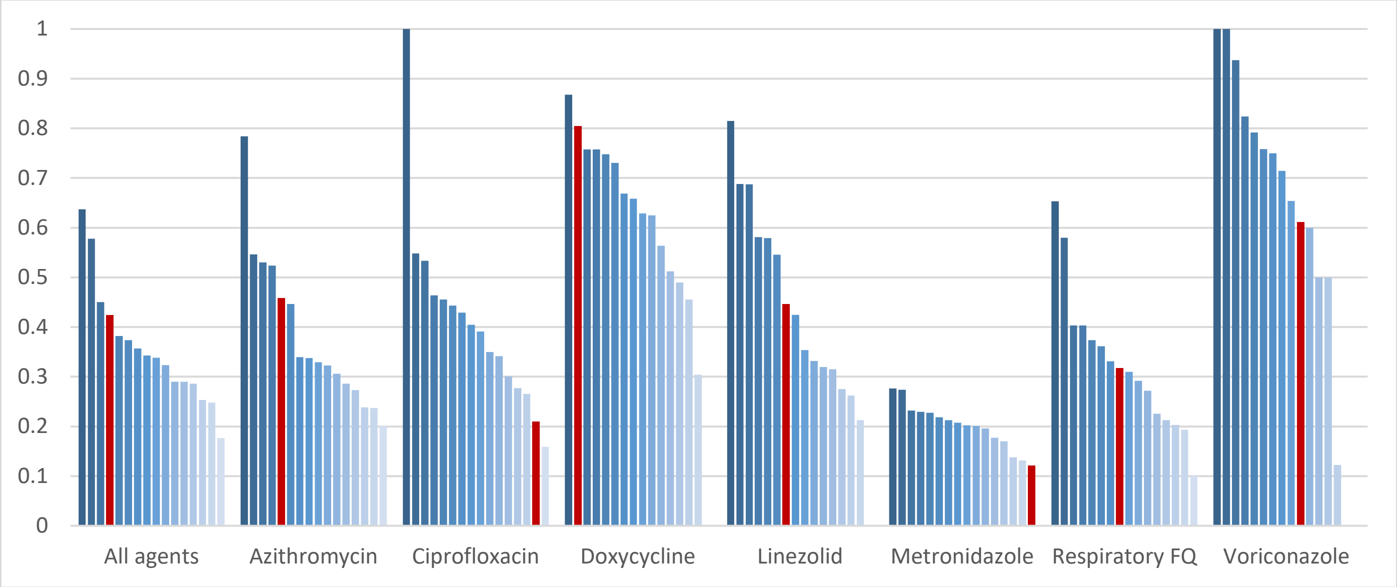

Red bars = Hospital Example; Respiratory FQ includes moxifloxacin and levofloxacin

Table 1. Proportion of digestive/total days of therapy and DASON benchmarks

	N	Digestive DOT, sum	Total DOT, sum	Digestive/total DOT, Mean (std)	Rank	DASON Expected Digestive/Total (Margin of Error)*	Below
All Agents	3725	5416	12332	0.42 (0.47)	4	0.41 (0.33-0.49)	
Azithromycin	846	914	1858	0.46 (0.47)	5	0.65 (0.32-0.98)	
Ciprofloxacin	533	330	1683	0.21 (0.38)	14	0.40 (0.34-0.46)	*
Doxycycline	934	2918	3739	0.81 (0.37)	2	0.59 (0.48-0.71)	
Linezolid	137	290	633	0.45 (0.48)	7	0.48 (0.31-0.65)	
Metronidazole	751	376	2714	0.12 (0.30)	16	0.19 (0.17-0.21)	*
Respiratory FQ	493	424	1453	0.32 (0.45)	8	0.34 (0.26-0.41)	
Voriconazole	31	164	252	0.61 (0.46)	10	0.74 (0.57-0.91)	

*Excludes your hospital and adjusts for the following variables: Length of stay, age, unit type, elixhauser score. "All agents" model also includes adjustment for agent.

Figure 2. Facility-wide digestive/total by Month (All Targeted Agents), 2016-2019

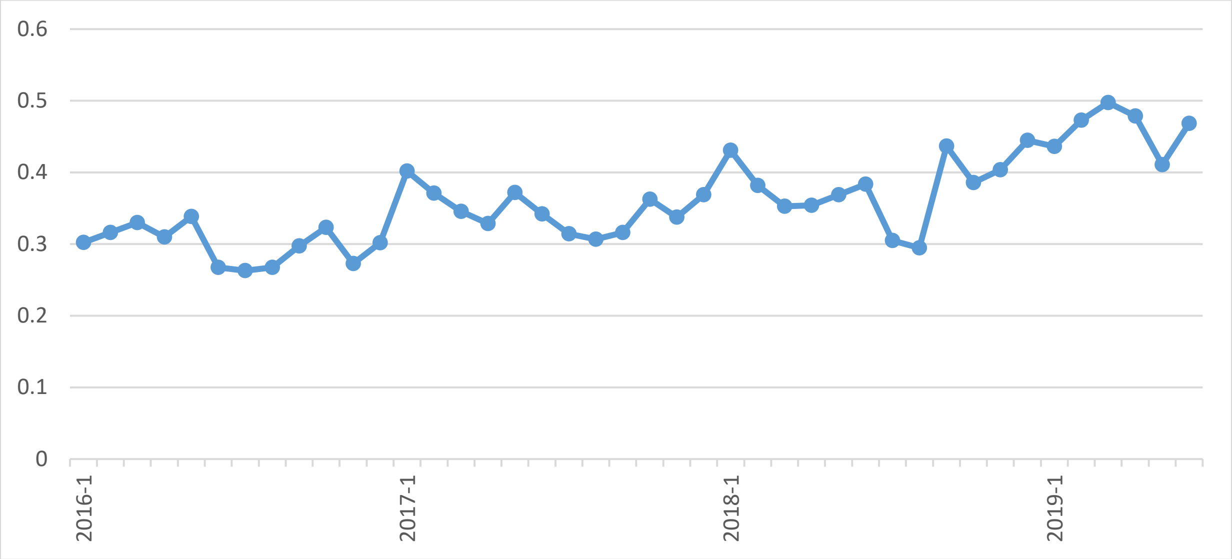

Table 2. Digestive/Total by Inpatient Unit,[a] Lowest 10

Unit Name	NHSN Unit Type	N	Mean Digestive/Total	Unit type DASON Mean[b]
ICU/CCU	Medical/Surgical Critical Care	625	0.24	0.18
5N	Surgical Ward	610	0.28	0.36
4N	ONC General Hematology/Oncology Ward	362	0.37	0.52
3N	Medical/Surgical Ward	928	0.48	0.37
1N	ONC General Hematology/Oncology Ward	425	0.50	0.52
2N	Telemetry Ward	775	0.61	0.45

[a] Inpatient Unit assigned on day 1 of the course of antimicrobial; [b] Mean Digestive/Total among patients in units of the same type, excluding your hospital's patients.
